# Assessing core capacities for addressing public health emergencies of international concern at designated points of entry in cameroon during the COVID-19 Pandemic

**DOI:** 10.1186/s12889-022-14614-7

**Published:** 2022-11-28

**Authors:** Viviane Fossouo Ndoungué, Arouna Njayou Ngapagna, Serge Agbo Kouadio, Raoul Djinguebey, Oumarou Gnigninanjouena, Sara Eyangoh, Georges Nguefack-Tsague, Hugues C. Nana Djeunga, Omer Njajou

**Affiliations:** 1 National Public Health Observatory, Ministry of Health, P.O. Box, 3051 Yaounde, Cameroon; 2 Université Des Montagnes, Bangangté, Cameroon; 3 Takling Deadly Disease in Africa, DAI, London, UK; 4 World Health Organization, Yaoundé, Cameroon; 5 Centre Pasteur, Yaoundé, Cameroon; 6 Faculty of Medicine and Biomedical Sciences, University of Yaounde I, Yaoundé, Cameroon; 7 Centre for Research On Filariasis and Other Tropical Diseases (CRFilMT), PO Box 5797, Yaoundé, Cameroon

**Keywords:** International health regulation, Points of entry, Port, Airport, Ground-crossing, Health emergencies, Core capacities, Cameroon

## Abstract

**Background:**

Points of Entry (POEs) are at the frontline for prevention, detection and response to international spread of diseases. The objective of this assessment was to ascertain the current level of existing International Health Regulations (IHR) core capacities of designated airports, ports and ground crossings in Cameroon and identify critical gaps for capacity building for prevention, early warning and response to public health threats including COVID-19.

**Methods:**

Data were collected from April to May 2020 in 5 designated POEs: Yaounde Nsimalen International Airport (YIA), Douala international Airport (DIA), Douala Autonomous Port (DAP), Garoua-Boulai ground crossing, Kye-Ossi ground crossing which were all selected for their high volume of passenger and goods traffic. The World Health Organization (WHO) assessment tool for core capacity requirements at designated airports, ports and ground crossings was used to collect data on three technical capacities: (i) communication and coordination, (ii) Capacities at all times and (iii) capacities to respond to Public Health Emergencies of International Concern (PHEIC).

**Results:**

All the investigated POEs scored below 50% of capacities in place. YIA recorded the highest percentage for all groups of capacities, coordination and communication and for core capacity at all times with a percentage of 42%, 58% and 32% respectively. For core capacity to respond to PHEIC, all the POEs recorded below 50%. The DAP and all ground crossings lacked trained personnel for inspection of conveyances. Only DIA had a public health emergency plan. There is no isolation/quarantine and transport capacity at the POEs.

**Conclusion:**

All POEs assessed did not meet IHR standards and need significant improvement to fulfill the IHR requirements. Unstructured communication channels between stakeholders make the implementation of IHR challenging. A coordination mechanism, with clear functions and structure, is necessary for well-coordinated response efforts to health emergencies at POEs. This assessment will serve as a baseline to inform planning and IHR implementation at designated POEs in Cameroon.

## Background

Although international transport, travel and trade contribute to economic development and welfare of populations, they may also pose public health risks. The increasing traffic at airports, ports and ground crossings, can play a significant role in the international spread of diseases through persons, conveyances and goods [1]. The Points of Entry (POEs) are challenging places to work as they involve the diversity of transportation of cargo and people from different areas of the world [2]. Cameroon experiences an average of forty measles outbreaks per year and those recorded in 2016 were associated to imported cases reference. The country also report at least one suspect case of cholera every year with about 44 cholera outbreaks recorded in the space of 44 years associated with imported cases [3]. The International Health Regulations (IHR) version 2005 is a global legal framework for public health security since it entered into force on 15 June 2007. It is legally binding in all signatories WHO State Parties including Cameroon. IHR aims at helping countries to prevent, protect against, control and respond to the international spread of diseases, while avoiding unnecessary interference with international traffic and trade, therefore saving lives and minimizing their impact on livelihoods [4]. In addition, IHR extends its scope by including the responsibility to respond to zoonotic diseases, food safety, chemical, and radiological hazards [5]. The (IHR, 2005) is also designed to reduce the risk of disease spread at international airports, ports and ground crossings [6]. Therefore, they are at the frontline for prevention, detection and response to diseases associated with international movements of people and goods [7]. Under the IHR, countries are required to designate airports, ports and may designate ground crossings to develop capacities for routine prevention, control measures, and response to events that may constitute a PHEIC provided in Annex 1 of the IHR and make necessary revisions to the national regulations to ensure provision of key sanitary, health services and facilities at designated POEs [6]. The purpose for providing POEs measures in the IHR is to reduce the international spread of diseases [8]. To this effect, numerous international airports, ports or ground crossings were designated by big State Parties, while in some small countries, a single airport and/or port was designated to manage travellers during public health crisis [9]. According to the IHR, State Parties are invited to establish response capacity and ensure that health measures are in place at designated airports, ports and ground crossings [1]. By doing so, the health of travellers and population is protected and transportation means are safe and hygienic. This will prevent unnecessary health-based restrictions on international travels and trade [10]. The Joint External Evaluation of the IHR helps to evaluating countries’ capacities required under the IHR in 19 technical areas including POEs and 48 indicators of which two are related to POEs [11]. Cameroon conducted the JEE in December 2017 and recorded no capacity (score 1 on a scale of 1 to 5) for both routine capacities at POEs and for effective public health response at POEs. One of the key recommendation was to officially designate the POEs and ensure at all designated POEs, access to appropriate medical services, including staff, equipment and diagnostic capabilities for the prompt examination and management of ill travelers [3]. To address this recommendation, Cameroon assigned 12 official POEs as designated including four international airports, three ports and five ground crossings to develop core public health capacities under the IHR. The last assessment of POEs in Cameroon was conducted a dozen years ago. The IHR capacities was addressed during this assessment in a summary manner in 2008 during the initial assessment of the IHR national capacity, with an emphasis on cross-border epidemiological surveillance. The country is now currently affected by the COVID-19 pandemic and at a certain point, a state of emergency was declared. As a result, the closure of borders was adopted as one of the containment measures to fight the pandemic. In order to be sure on the real resources and capacities of POEs to meet IHR requirements in Cameroun, an assessment was needed to appreciate the current status of IHR capacities at POEs. The objective of this assessment was to ascertain the current level of existing IHR core capacities of designated airports, ports and ground crossings in Cameroon and identify critical gaps for capacity building for prevention, early warning and response to public health threats including COVID-19.

## Methods

### Study design and setting

**Fig. 1:**
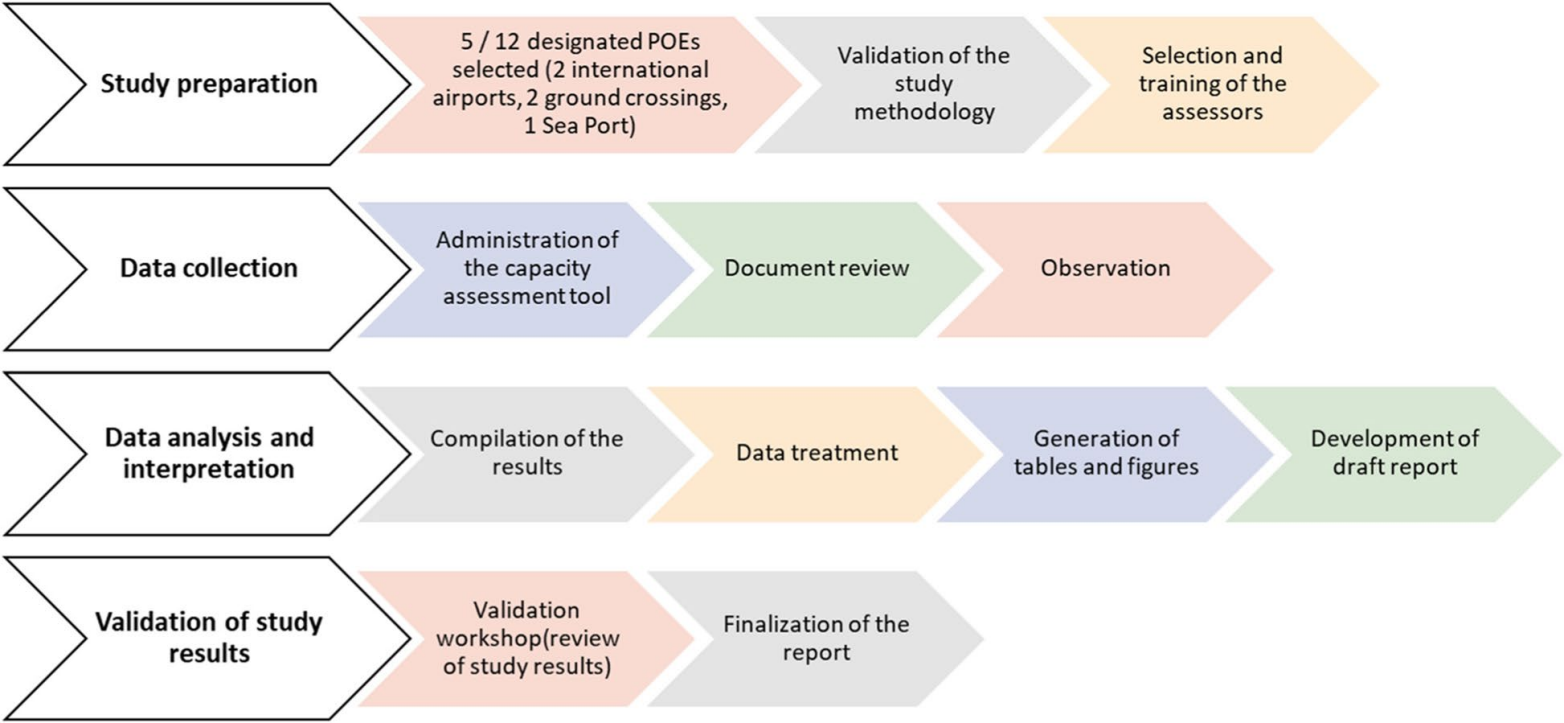
Methodological flow chart

A cross sectional study was conducted. Data were collected for 3 days during a period of April to May 2020. Five out the 12 designated POEs in Cameroon of which two international airports (Yaounde Nsimalen and Douala), one see port (Douala autonomous port), and two ground crossings (Garoua-Boulai and Kye-Ossi) were selected and enrolled in the assessment. The inclusion criteria were: (i) be a one of the designated POEs in Cameroon, (ii) the high volume and frequency of international traffic, (iii) the population density around the POE, (iv) the epidemiological and risks profiles of the POE, (v) neighboring countries, (vi) existing health care facilities in less than five kilometersradius and (vii) refusing to participate in the study was the exclusion criteria. The study methodological flow chart is summarized in Fig. 1 below.

### Profile of the assessors

A multidisciplinary task force of six people was constituted to perform the assessment at the selected POEs. The members were made of public health specialists from human and animal health, university lecturer, Epidemiology, scientific research, social sciences expert with a good knowledge on public health issues including health emergencies and IHR. A supervisor from Ministry of health was designated to oversee the process.

### Preparation of the assessment

The task force conducted three preparatory meetings to train surveyors on the tool, develop a strategy and timeframes for timely completion of the assessment and address any issue arising. Upon arrival in the administrative region where the POE to be assessed is located, a courtesy call was paid to the Regional Delegate for Public Health (RDPH) to introduce the assessment team and present the objectives of the mission.

### Assessment tool

The assessment tool for core capacity requirements at designated POEs provided by WHO in 2009 was used as the data collection tool (accessible at https://apps.who.int/iris/handle/10665/70839) [6]. In this tool, the capacity requirements were defined by 95 assessing indicators, which enable state parties to identify existing capacities or potential gaps, along with the formulation of plans of action that address the capacities that need to be improved. The 95 assessing indicators are being utilized since 2009 in all WHO Member states to evaluate the core capacities of POEs. Therefore, it is reliable because it is consistent over time. The external validly is adequate because the outcome of this study can be expected if we apply to other settings. Concerning the internal validity, the investigators were trained to understand the tool and the data collection was monitored to ensure that the tool protocol as defined by WHO is well-conducted [6]. The tool is divided into 3 modules: (i) communication and coordination framework among various stakeholders, (ii) capacities necessary at all times also called “routine capacities” and (iii) capacities for responding to Public Health Emergencies of International Concern (PHEIC). For each indicator, the assessor had only 3 possible answers in a drop-down box: Fully implemented(Y), Not implemented (N) and partially implemented (Partial). Some capacities required only the presence or absence of certain criteria, while others were comprehensively analyzed based on the on-site performance and document review. But if the assessor taught that the answer to the question applies to both “Y” and “Partial” or “N” and “Partial”, he/she was systematically choosing the most accurate answer of the two. To complete the assessment, the assessor was inputting comments and suggestions in the summary worksheet, highlighting areas where clarification of regulatory requirements is needed or where improvements are needed to attain full implementation of IHR core capacities.

In addition to using the electronic and paper version of the tool, all assessors had a notebook and a (digital) camera for documentation.

### Training of the assessors on the utilization of the tool

The assessors were trained in the proper utilization of the data collection tool and a practical exercise was conducted at the Yaounde Nsimalen international airport on 27 March 2020. At the end of this exercise, the meaning of the questions was clarified to assessors. The stakeholders who participated in this exercise did not participate in the assessment.

### Data collection

The validation approach, we have followed up external, internal and process validation as indicated by WHO [6]. Indeed, the tool was administered to the health authority and his team at the POEs, then non health stakeholders were met individually. The stakeholders and competent authorities of the POE agreed to undertake this assessment. The assessors therefore observed and recorded information on paper-based data collection tools while the staff was on-the-job. The feedback on the strengths, weaknesses and plans for future improvements was provided to the different regional delegates for public health and to airport authorities, port authority and sub-divisional officers.

### Document review

The assessors reviewed all the guidelines, Procedures, management documents, Memorandum of Understanding, protocols that mentioned in the WHO assessment tool.

### Field visit

As required in the “assessment tool for core capacity requirements at designated POEs https://apps.who.int/iris/handle/10665/70839 [6], assessors walked around the areas related to the public health operation outlined in the checklist and completed each area on the checklist by writing down clearly specified comments which reflected the rationale in the comments space of the sheet. During the field visit, the assessors documented the assessment by taking pictures of the POE, staff in action, facility, equipment, operation, etc. These pictures were useful to illustrate and explain the core capacity conditions at the POE in the final report.

### Completing the core capacity assessment tool

The assessors were collecting data using the paper-based forms which were later uploaded into the WHO Excel sheet File for analysis. Once the visual evaluation of the POE was completed, the assessors then completed the strengths, weaknesses and plans for future improvements and discussed with the stakeholders for validation.

### Stakeholders assessed

The assessment process was multisectoral and multi-agency. The stakeholders involved were selected based on their implication in POEs activities. These stakeholders included the Ministry of Forestry and Wildlife, Ministry of Livestock, Fisheries and Animal Industries, Ministry of Environment, Nature Protection and Sustainable Development, Ministry of Territorial Administration, Gendarmerie, Police, Civil Aviation, Cameroon Airport, Port Authority, Airport Authority, Customs services and, Sub-divisional officer. The representatives from each institution were selected based on their experience and involvement in POEs activities. Newly appointed staff and intern were excluded.

### Data management

#### Data validation

A three-day workshop was conducted from 14 to 16 October 2020 to validate data collected during the field assessment. About 35 participants from sectors operating at the POEs and their respective authorities at the national level gathered to validate the data collected during the field visits. All the answers in the excel spread sheet were reviewed, discussed and approved based on consensus of stakeholders by hand raising.

#### Data analysis

Data analysis was conducted according to the guidance provided the “assessment tool for core capacity requirements at designated POEs (https://apps.who.int/iris/handle/10665/70839)(6). When the performance for each group of core capacities was determined and uploaded into the Excel Spread Sheet by the assessor, calculations of groups of core capacities were automatically generated. The calculations are based on the following principles:

- Answering “Yes” gives 1 point or 100% to the question.
- Answering “No” gives 0 or 0% points to the question.
- Answering “partially” gives 0.5 or 50 points to the question.
  The results were expressed with different background colours ranging from red to green:
- Red: Below 50%—significant improvement needed.
- Yellow: Between 50 and 80%—some improvement needed.
- Green: Above 80%—POE is fairly consistent with the requirements of IHR Annex 1.

The final results were also reflected in numerical and graphical form in the “Summary” worksheet, with space provided for assessors to input their comments and captured evidence (photographs, video clips and observations).

### Administrative authorization

A letter of authorization was obtained from the Minister of Public Health to carry out the study and administrative letters were sent to staffs and authorities to be interviewed at the POEs. All methods were performed in accordance with the relevant guidelines and regulations.

### Limitations

The PoEs included in this study were not selected randomly. However, the 5 POEs included can be representative of the 12 designated POEs in Cameroon. Therefore, the results of our study can be generalized to reflect the IHR capacities of POEs in Cameroon.

## Results

Overall, all the assessed POEs scored below 50% and therefore need significant improvement to meet the IHR core capacities required at POEs. YIA recorded the highest average score for all groups of capacities (42%), followed by the DIA (36%), the DAP (36%), the KOGC (36%). The GBGC recorded the lowest average score for all groups of capacity (28%) (Table 1).

**Table 1:** Total score and score for coordination, capacity at all times and capacity to respond to PHEIC in the 5 POEs assessed in Cameroon, 2020

	Yaounde Nsimalen international airport	Douala international airport	Douala autonomous port	Kye-0ssi ground crossing	Garoua-Boulai ground crossing
**All groups of core capacities**					
Coordination and communication	58%	44%	44%	44%	39%
Core capacities at all times	32%	28%	28%	25%	13%
Core capacities for responding to PHEICs	35%	36%	36%	38%	32%
** Average score**	**42%**	**36%**	**36%**	**36%**	**28%**
**Coordination and communication**					
International communication link with competent authorities at other points of entry	0%	0%	0%	0%	50%
National communication link between competent authorities at points of entry and health authorities at local, intermediate and national levels	75%	50%	50%	50%	50%
Direct operational link with other senior health officials	50%	50%	50%	50%	50%
Communication link with conveyance operators	50%	100%	100%	50%	50%
Communication link with travellers for health related information	50%	50%	50%	50%	50%
Communication link with service providers	50%	50%	50%	50%	0%
Assessment of all reports of urgent events within 24 h	100%	50%	50%	50%	50%
Communication mechanism for the dissemination of information and recommendations received from WHO	100%	100%	100%	50%	50%
Procedures and legal and administrative provisions to conduct inspections	50%	50%	50%	50%	0%
**Core capacities at all times**					
Access to medical service	50%	67%	67%	50%	42%
Transport of ill travelers	50%	38%	50%	25%	38%
Trained personnel for inspection	50%	53%	42%	45%	10%
Safe environment for travelers	45%	55%	17%	14%	11%
Vector control program	25%	13%	38%	13%	0%
Special capacities	33%	0%	50%	50%	0%
**Core capacities for responding to PHEICs**					
Public health emergency contingency plan	50%	17%	50%	17%	0%
Assessment/care for affected travelers/ animals	58%	17%	17%	33%	33%
Space for interview suspect/affected persons	33%	33%	50%	50%	33%
Quarantine of suspect travelers	38%	50%	50%	50%	50%
Public health measures	25%	38%	25%	38%	25%
Entry or exit controls	0%	50%	0%	50%	50%
Trained personnel on PPE use	42%	50%	50%	25%	33%

Regarding coordination and communication capacity, the YIA recorded the highest average score (58%). All the other four POEs recorded less than 50% average score for this capacity suggesting a significant improvement to fulfill the (IHR,2005) requirements (Table 1). The GBGC recorded the lowest score for this group of capacity (39%). The findings highlighted some strengths including [1] good collaboration between the border health post and the public hospitals of the region and availability of directory of hospitals, clinics and health centers, [2] good communication link between the POEs and health authorities of the region following the command chain even though this should be extended to private health facilities, [3] good communication link between the border health posts and the national IHR focal point via an internal communication network for the transmission of information and recommendations from WHO. The weaknesses identified in all the POEs assessed were related to: [1] absence of an international communication network with competent authorities of the destination POEs, [2] poor communication between the different sectors operating at the POEs, [3] absence of legal procedures for inspections of conveyances. Specifically, the DIA, DAP and GBGC do not have the necessary means and resources to assess reports of urgent events at the point of entry within 24 h and only communicate with service providers where possible. In addition, communication with transport operators is partially satisfactory at the APD as compared to KOGC and GBGC where this communication link facilitates the assessment of urgent reports on public health events within 24 h. Interestingly, the KOGC border health post is a member of WhatsApp group that brings together officials from the Gabonese, Equatorial Guinean and Cameroonian POEs. This is a significant development in terms of cross borders communication and interventions.

As for core capacity at all times, the YIA recorded the highest average score (32%) followed by the DIA and the DAP which recorded a score of 28% each. The GBGC recorded the lowest score for this group of capacity (13%) (Table 1). The strengths reported by the assessors to improve capacity at all times at all POEs assessed included [1] availability of a space for assessment and interview of sick/suspects travelers and capacity for control of immunization status of travelers, [2] basic knowledge on the use of Personal Protection Equipment (PPE) by health staff. The weaknesses identified were mainly as a result of [1] inadequate infrastructures and equipment for the implementation of IHR requirements, [2] absence of a space for isolation/quarantine of sick/suspect travelers and absence of transportation means to transfer sick/suspect travelers to appropriate health facilities except for the YIA where one ambulance was available, [3] absence of a program for vectors and reservoir control and no trained personnel to undertake the vector disease surveillance, [4] absence of a plan for air quality control and procedures/plans for inspection of conveyances, application of public health measures, safe management of human remains, delivery of free practice at all the POEs.

With regard to core capacity for responding to PHEIC, all the POEs recorded a score less than 50% indicating significant improvement to achieve the required IHR capacities. The KOGC recorded the highest average score (38%) followed by the DIA and the DAP which recorded an average score of 36% each (Table 1). The major strengths highlighted were [1] the availability of an emergency plan at the airports, [2] availability of a space to interview sick/suspect travelers, [3] availability of trained veterinarians and phytosanitary staff at all POEs. The weaknesses identified at all POEs were mainly due to [1] absence of a risk mapping profiling, public health emergency plan and hazard specific plans, [2] absence of simulation exercises to test the developed plans, [3] absence of capacity for isolation or quarantine of sick travelers during public health emergencies, [4] no updated training needs and absence of a training plan to continuously build airport staff capacity on implementation of public health measures at the POE, [5] absence of a program for sanitary inspection of the POE facilities and conveyances (Table 1).

With regards to the competent authority at POEs in Cameroon, five key ministries are in charge of application of health measures to prevent the international spread of diseases. However, other ministries such as the customs, the ministry of trade and ministry of defense provide their support as requested by competent authorities (Table 2).

**Table 2:** Competent authorities at POEs and competency areas in Cameroon, 2020

Competent authorities at POEs	**Areas of competency**
Ministry of Public Health	Information of travelers about health risks, preventive measures and facilities for management, -Ensuring sanitary control of travelers, -Ensuring cross-border epidemiological surveillance, -Ensuring transfer and protection of sick travelers, -Implementation of sanitary measures in the POE (desinsectization, deratization), sanitary inspection of conveyances, containers and cargo, -Control of medicines and psychotropic drugs
Ministry of Livestock, Fisheries and Animal Industries	-Sanitary inspection of livestock, animal or fish products and their derivatives unfit for consumption, -Quarantine of suspect animals, -Seizure/destruction of animal or fish products and their derivatives unfit for consumption, -Issuance of sanitary travel permits for livestock and animal products travelling -Cross-border epidemiological surveillance, -Sanitary inspection of conveyances, control of transport and storage conditions of veterinary products
Ministry of Forestry and Wildlife	Control of forestry and wildlife products
Ministry of agriculture and rural development:	-Control of agricultural and phytosanitary products, -Issuing of travel permits for controlled products, -Verification of hygiene and safety of foodstuffs for cross-border movement
Ministry of Environment, Nature Protection and Sustainable Development	Environmental inspection of conveyances and cargo
Customs	-Provide support to other sectors, especially in the enforcement of specific laws and regulations, -Control of radiological cargo
Ministry of Trade	Ensures compliance with the requirements of technical regulations or standards of goods
Ministry of Defense (fire brigade, army, air force, navy)	Ensures the respect of order and compliance by travelers and delimits the security areas
General Delegation of National Security	-Control of identity, -Provide support to other sectors to ensure security and order, -Issue of "shore pass" or visa

## Discussion

This study sets to assess the existing capacities at designated POEs in Cameroon. We found that overall, the POEs assessed in Cameroon need significant improvement (score below 50%) to fulfill the IHR requirements. Cameroun POE therefore has longer way to go compared to other developing countries such as Uruguay [12] and India [13] where IHR capacities need just few improvements to meet IHR standards. This poor performance of POEs in Cameroon can be attributed to the unilateral designation of the POEs by the Ministry of Public Health. The designation of the POEs based on consensus, bringing together the stakeholders involved in the implementation of the core capacities and not only from the health sector has greatly contributed in building IHR capacities at the POEs in Taiwan [14]. Besides, the animal health sector also has an entry point at the border, it is therefore important to also create a direct link between human and animal health sectors which will lead to the establishment of a single pattern for these entities to work in a one health approach from now on.

### Coordination and communication

Good coordination networks are the best solution to the implementation of policy, program, or project [15]. Significant improvement is needed to strengthen this capacity in Cameroon for all types of POEs(score below 50%) while in India, findings show that some improvement was needed to build this capacity in ground crossings(76%) [13]. Our findings highlighted a strong correlation between “Coordination and communication” and “core capacities at all time” and between “Core capacities at all times” and “Core capacities for responding to PHEICs. This corroborates the findings of a study examining the implementation structure of the National Environmental Action Plan in Madagascar and how coordination problems were manifested. The findings were that a well-organized and coordinated network help actors in the policy implementation and in achieving the organizations’ end results [15]. We noted that a good communication link existed between the port health officer and health authorities at regional and national level. However, there is need to streamline the coordination amongst various departments under the Ministry of Health (MoH) involved in the implementation of different components of the IHR at the POEs. In Cameroon, about four departments under the MoH intervene at the POEs (IHR National Focal Point (NFP), Disease control department, immunization department, department of health care organization and health technology). These departments operate in silos and their interventions and efforts at POEs are not coordinated, leading to limited impact on building IHR with differences seen in the prioritization of POEs for intervention. The situation was also observed in Tanzania where the three sections of the MoH involved in IHR POEs had separate uncoordinated plans with regard to its implementation [2]. Inappropriate and infrequent coordination during the implementation of any policy may lead to conflicts of authority within the organization partly due to lack of clear understanding of stakeholders’ roles and responsibilities [15]. Unstructured communication channels between IHR-NFP, other departments under the MoH, WHO and other sectors complicate the implementation of IHR [2]. Therefore, while developing the POEs core capacities, it would be more efficient if consensus and resource integration are achieved in advance, at the national level, bringing together all the competent authorities under a single program called “IHR POE program” in Taiwan [14] and “border health strategy” in Nigeria, Benin, and Togo [16]. Port health officers at POEs in Cameroon use a WhatsApp group to directly communicate with the IHR-NFP for information sharing on alerts and potential PHEIC reported in other countries or in one of the POEs in the country. Cross border surveillance data, official communications from the IHR-NFP as well as urgent guidelines from WHO are also shared in this platform. Access to the internet improves a timely and reliable communication and information sharing. Health care workers in Julius Nyerere International Airport (JNIA) in Tanzania preferred the MoH website to be used as the official source of information for communicating alerts. However, communication facilities such as computers, printers, fax machines, and internet connection are not consistently available [2].

All POEs assessed recorded need significant improvement to fully achieve the capacity for coordination and communication at the POEs. It has been demonstrated that meeting IHR requirements at a POEs is a common challenge involving multisectoral engagement and communication. A coordination mechanism, with clear functions and structure, is therefore necessary for a well-coordinated response efforts to health emergencies arising at POEs [14]. An agreed protocol, which clarifies the strategies, timeline, and multidisciplinary/multisectoral duties, is essential for an effective coordinated efforts for health emergencies capacities for preparedness and response at POEs [14]. Some countries developed an inter departmental network or platform with clear functions and structure, at either the central or POE level, in order to facilitate the establishment of the IHR core capacities [14]. After the SARS outbreak in 2003, Taiwan established a multi stakeholders network called the “port sanitary group’” which successfully work to achieve efficient information sharing, policy declaration and coordination of public health measures carried out at the POEs during the H1N1 pandemic in 2009 [14]. Lesson learnt from this experience lead the country to establish a “POE Taskforce” at designated ports and airports to facilitate the fulfillment of the IHR core capacity requirements [14]. Some stakeholders in India highlighted the importance of sharing information related to public health events and suggested the need for cross border meetings at ground crossings. They further indicated that building and maintaining these open lines of communication between neighbouring nations are critical to effectively respond to disease and environmental issues at cross borders level. This allows stakeholders to exchange their insights, build relationships and identify areas for future joint improvements that can be incorporated into guidelines for IHR implementation, especially at ground crossings [13]. This approach may seem very useful to Cameroun which is circled by six countries and still struggling to establish solid functioning POE system.

### Core capacities at all times

Our findings shown that significant improvement is required to acquire the IHR routine capacities at POEs while in India, they were fairly consistent with IHR requirements at ground crossings(83%) [13]. Access to medical care at POEs, especially airports is generally provided. In Cameroon, all the two designated airports assessed possess the capacity to interview sick/suspect travellers and capacity for control of immunization status of travelers. This corroborates with an assessment conducted at the JNIA in Tanzania where heath care workers reported that the airport had provisions for the control of the required HIR documents, especially the international certificate of vaccination against yellow fever, or other prophylaxis and Aircraft General Declaration [2]. Low understanding of the HIR requirements have been identified as one of the challenge of IHR implementation at the JNIA. Indeed, health care workers had little information or understanding and were unsure about the objectives of IHR [2]. In the contrary, in India, the stakeholders reported a good understanding of the HIR implementation and the measures that need to be taken during routine and emergencies [13]. The port and ground crossing in Cameroon need significant improvement with regard to availability of trained personnel for inspection of conveyances. This finding correlates with a study for assessment of training need in major European POEs where ports scored routine vessel inspection as of high importance and expressed a high training need for routine inspections as well [17]. There is need to establish a program for vector control and train the personnel in all the POEs in Cameroun meanwhile monitoring of vector control were reported as important activities that are regularly conducted in ground crossings in India, [13] and in airports in Taiwan [18]. In addition, we also found that there was also a need to develop a plan for air quality control to ensure to ensure a safe environment and apply control measures for potential risks from air quality. Similar results were obtained in India after an assessment of ground crossings [13].

### Core capacities for responding to PHEIC

We found that there was significant improvement needed to build the capacity for responding to PHEICs in the all types of POEs in Cameroon compare to India were some improvement is needed(Score 68%) [13] Apart from the DIA, none of the POEs assessed had a public health emergency plan compare to the JNIA which, even though non designated, developed a public health emergency contingency plan to enable JNIA authorities to prepare and respond rapidly to any emergencies. However, the plan is not implemented due to insufficient budget allocation [2]. The findings of a study which assessed global public health surveillance under the HIR reported similar findings indicating that adequate resources mobilization to implement HIR in poorly resourced countries is challenging [13, 19]. For countries to have a successful implementation of IHR and sustainable capacities at POEs, there is need for mobilizing and allocating adequate resources such as budget, skilled personnel, appropriate technology and infrastructures including offices and transport facilities [2]. When the plan is in place, testing it varies depending on countries level of awareness. Studies conducted in Taiwan indicate that the emergency response system regularly undergoes exercises through full scale simulation exercises [18] whereas stakeholders in India reported the absence of regular simulation exercises to familiarize key sectors with the content of the plan and respective roles and functions within it. They suggested having an emergency operations centre that can help meet the requirement for routine reporting systems and facilitate early response [13]. There are no isolation/quarantine capacity at the POEs in Cameroon for examining suspected or ill travelers. Sick travellers are transferred to designated health care facilities using public transports. The ambulance of the YIA is old and poorly maintained and in DIA, there is an agreement between the port health and a private health care facility to request for their ambulance when needed. Similar situation was observed in the JNIA where it was observed that isolation rooms did not have the required equipment for service provision. As a result, sick/suspects passengers were transferred to selected private hospitals in the city with proper and well-equipped isolation rooms. In addition, it was also reported that the only ambulance available was not reliable as a result of poor maintenance making it difficult to transport suspects to the nearby health facilities [2].

The findings of this study shown that in Cameroon, the implementation of public health measures at POEs need significant improvement. This topic was identified as of high training need by respondents in the major POEs in Europe. In addition, other publications indicate that several POEs experienced enormous challenges to handle cruise ships with COVID-19 cases on board [20, 21]and implement public health measures at airports and on land-borders, resulting in closure of borders as a containment measure for the COVID-19 pandemic [22].

Significant improvement needed in YIA and ground crossings and some improvement needed in DIA and DAP with regard to the availability of trained personnel on proper use of personal protective equipment. These findings correlate with a study conducted in 50 POEs from 19 European countries to assess training needs for infectious disease management at major POEs where the use of personal protective equipment both in routine and response situation recorded the highest mean score of high importance out of the twenty-four proposed topics. Simulation exercises were the most preferred method of training for practical skills such as the use of personal protective equipment and the handling of ill persons [17].

### Competent authority

The identification of the competent authority of the POE has always been debated among the different stakeholders [14]. This has been the case for Cameroon as well. The discussions started at the airport level during the trainings on IHR implementation. The initial understanding was that the Cameroon Airports, which is a company in charge of the administration and management of airports in Cameroon would be the competent authority. However, the IHR's definition of the competent authority highlights that this authority should be responsible for the implementation of health measures [4]. The consensus in Cameroon like in Taiwan was therefore that the competent authority will vary depending on the type of health measures to be implemented [14]. In addition, the interest in formalising this role through an administrative act was not supported by the stakeholders in Cameroon because the presence of the sectors in charge of the implementation of the different health measures in the POE gives them full power to operate according to their mandate.

### Implications for the COVID-19 response at the POEs in Cameroon

After declaration of the COVID-19 as a PHEIC by WHO, countries were classified according to risk to the level of risk of importation or spread of the COVID-19 disease. According to the WHO risk analysis, the risk of spread was very high in China and high for all other countries [23]. The national health authorities in Cameroon also conducted a risk assessment. The localities with the highest risk of importation in Cameroon are those with points of entry especially Douala and Yaounde of the large flow of international trade through the YIA, DIA and the DAP. Likelihood of occurrence was therefore graded “likely”. If a single case of COVID-19 is recorded in the country, this will lead to major impact on the population with major disruptions to activities and services. In addition, many additional control measures will be required with a significant increase in costs. The consequences were rated as “major”. Based on this analysis, it is likely that a case of COVID-19 will be imported into Cameroon and that this will have major consequences. The authorities therefore concluded that the risk of importing a case of COVID-19 into the country was high. The two first confirmed cases were reported in Cameroon on March, 6^th^ 2020. The first case was a 58-year-old man arriving from France and entered Cameroon via the YIA on 24 February 2020, without presenting any symptoms. He consulted on 5^th^ March for fever and fatigue evolving since 25^th^ February 2020 and was confirmed positive for COVID-19. The second case was a 30-year-old woman residing in Yaounde, with an epidemiological link to the first case and identified during contacts tracing. The first case generated 162 contacts of which 40 were high risk contacts and the 2^nd^ case generated 14 contacts. All the 81 passengers who arrived with the same flight were all traced and monitored. Following this situation, Cameroon implemented a sets of public health and social measures as containment measures including travel restrictions through POEs within a week of the first case [24]. If the country wants to win the battle against the COVID-19 pandemic and prevent the importation of more cases into the country, critical gaps identified during this assessment should be addressed as a matter of priority and maintained even after resuming international travels. WHO has recommended four main measures to implement at POEs for proper management of ill traveler’s in the context of the current COVID-19 pandemic including [1] detection of ill travelers, [2]interview of ill traveler’s to identify symptoms/exposure to the SARS-Cov 2 virus, [3] reporting suspected COVID-19 cases, [4] isolation, initial case management and referral of those with suspected COVID-19 infection [25]. An appropriate number of personnel should be assigned at designated POEs for the detection of potential cases/suspects, depending on the volume of travelers. This staff should be trained on identification of COVID-19 suspect cases and on the correct use of PPEs to protect themselves and maintaining more than 1 m between themselves and travelers at all times. Handheld no-touch (Infrared) thermometers or thermal cameras should be placed at the strategic areas through which travelers enter into the country and used to ascertain a traveler’s temperature. As many cases of COVID-19 are asymptomatic, health care workers should be trained on visual observation to identify suspects exhibiting signs suggestive of COVID-19 disease as they pass through the entry point. In the literature, the authors' opinions on the effectiveness of entry/exit screening are divergent. One hand, some studies found that percentages of confirmed cases identified out of the total numbers of travellers that passed through entry screening measures in various countries worldwide for Influenza Pandemic (H1N1), Severe Acute Respiratory Syndrome and Ebola virus disease in West Africa were zero or extremely low [26, 27, 28]. On the other hand, a success story is reported on the effectiveness of the Taiwan border entry screening program in detecting the first Zika case in the country at the international airport in 2016 [18].

All authors however are unanimous on its positive effects including discouraging travel of ill persons, raising awareness, maintaining international traffic from/to the affected areas [27] and reduces community transmission risk and provides opportunities for national prevention and preparedness efforts and risk communication [18]. Our findings revealed that the port health did not have the capacity to isolate/quarantine ill or suspect travelers, the country should identify or set up a structure close to the POE where ill travelers can be referred to wait for an interview. This structure should also have the capacity to isolate travelers who, after interview, are suspected of having COVID-19 disease, while waiting for transport to a dedicated healthcare facility. Given the strong communication link between the POEs and health care facilities in the region, specific arrangements should be made with local healthcare facilities for prompt referral of COVID-19 cases. In addition, a long-term quarantine facility should be identified/set up separate from the POE for accommodate of large number of contacts. With regards to commodities, conduct a need assessment, procure and ensure a sustained supply of alcohol-based hand rub or soap and water, medical masks, waste bins with liners and lids, cleaning supplies, chairs, and beds in the isolation areas. To ensure a standardized management of case/contacts, guidelines, and procedures (interview, referral, cleaning etc.) should be developed and made available at all POEs. This study also revealed there was no capacity for transfer of ill/suspect travelers to dedicated health care facilities. Transportation companies capable of applying the recommended measures for cleaning and disinfection should be hired to transfer suspected cases to the identified healthcare facilities. A clear communication mechanism should be established between port health officers, representatives of the national civil aviation and maritime authorities, conveyance operators and POE operators and between port health officers and national health surveillance systems to report suspected COVID-19 cases identified at POEs. The HIR health documents (health section of the aircraft General Declaration form, Maritime Declaration of Health) should be submitted to the port health officers to assist in the collection of information about ill travelers on board with clinical signs or symptoms suggestive of COVID-19 disease. ethical requirements including optimizing isolation conditions, giving consideration to the quality of live to those who are isolated is essential to reduce social and economic disparities [20].

## Conclusion

The findings of this study revealed that, overall, there is significant improvement needed in Cameroon to develop and sustain core (HIR, 2005) capacities at POEs. There is need for strengthening coordination and communication as a strong link have been identified between this capacity and capacity at all times. Absence of a vector control and inspection program has been highlighted as a weakness across all types of POEs. To effectively respond to PHEICs, there is need to develop public health contingency plans based on a risk profiling and get them regularly tested through simulation exercises. In the era of the current COVID-19 pandemic, efforts should be directed to training port health officers and providing them with material for screening, personal protection, disinfection of environment. In addition, there in need to identify facility for isolation/quarantine of suspect travelers and strengthen the system for the transfer of travelers to designated facilities for appropriate management. This study can serve as a baseline for HIR capacity assessment at major designated POEs in Cameroon. The progress can be evaluated after the COVID-19 pandemic is declared over to appreciate the possible contribution of outbreaks on building IHR capacities at POEs.

Recommendations:

1. Build consensus between all the competent authorities and integrate resources under a single “IHR POE Program” at the national level to leverage on the efforts of all the stakeholders.
2. Establish an agreed protocol, which clarifies the strategies, timeline, and multidisciplinary/multisectoral duties for an effective coordinated efforts for health emergencies capacities for preparedness and response at POEs.
3. Conduct regular cross border meetings at ground crossings for sharing information related to public health events to effectively respond to disease and environmental issues at cross borders level.
4. Establish a vector control and inspection program for an adequate and safe inspection of conveyances across all types of POEs.
5. Develop public health contingency plans based on a risk profiling and conduct regular simulation exercises.
6. Train port health officers and provide them with material for screening, personal protection, disinfection of environment.
7. Identify facilities for isolation/quarantine of suspect travelers and strengthen the system for the transfer of travelers to designated facilities for appropriate management.

## Data Availability

The datasets used and analyzed during the current study are available from the corresponding author on reasonable request.

## Abbreviations

COVID-19: Corona Virus Disease 2019
DAP: Douala Autonomous Port
DIA: Douala International Airport
GBGC: Garoua-Boulai Ground Crossing
IHR: International Health Regulation
IHR-NFP: International Health Regulation-National Focal Point
JNIA: Julius Nyerere International Airport
KOGC: Kye-Ossi Ground Crossing
MoH: Ministry of Health
POE: Point of Entry
PHEIC: Public Health Emergencies of International Concern
RDPH: Regional Delegate for Public Health
WHO: World Health Organization
YIA: Yaounde-Nsimalen International Airport
